# A Severe Case of Lymphomatoid Papulosis Type E Successfully Treated with Interferon-Alfa 2a

**DOI:** 10.1155/2017/3194738

**Published:** 2017-04-30

**Authors:** Aslı Bilgiç Temel, Betül Unal, Hatice Erdi Şanlı, Şeniz Duygulu, Soner Uzun

**Affiliations:** ^1^Dermatology and Venereology Department, Akdeniz University Faculty of Medicine, Antalya, Turkey; ^2^Pathology Department, Akdeniz University Faculty of Medicine, Antalya, Turkey; ^3^Dermatology and Venereology Department, Ankara University Faculty of Medicine, Ankara, Turkey; ^4^Dermatology and Venereology Department, Pamukkale University Faculty of Medicine, Denizli, Turkey

## Abstract

Lymphomatoid papulosis (LyP) is a benign papulonodular skin eruption with histologic features of malignant lymphoma. A new variant of LyP which was termed “type E” was recently described with similar clinical and histological features to angiocentric and angiodestructive T-cell lymphoma. LyP type E is characterized with recurrent papulonodular lesions which rapidly turn into hemorrhagic necrotic ulcers and spontaneous regression by leaving a scar. None of the available treatment modalities affects the natural course of LyP. For therapy various modalities have been used such as topical and systemic steroids, PUVA, methotrexate, bexarotene, and IFN alfa-2b. Here we present a severe and devastating case with a very rare variant of LyP type E, which is, to our knowledge, the first case successfully treated with IFN alfa-2a. Now disease has been maintaining its remission status for six months.

## 1. Introduction

Lymphomatoid papulosis (LyP) is a benign papulonodular skin eruption with histologic features of malignant lymphoma. It has been listed as a primary, cutaneous, CD30 (+) lymphoproliferative disorder in the current World Health Organization (WHO) and European Organization for Research and Treatment of Cancer (EORTC) classification [1]. Histopathologically, there are well-known 4 LyP types (type A-wedge-shaped infiltrate containing eosinophils and histiocytes, type B-epidermotropism, resembling mycosis fungoides, type C-cohesive sheets of CD30 (+) cells, resembling anaplastic large cell lymphoma, and type D-CD8 (+), resembling primary cutaneous aggressive epidermotropic CD8 (+) cytotoxic T-cell lymphoma) [2]. A new variant of LyP which was termed “type E” by Kempf et al. was recently described with similar clinical and histological features to angiocentric and angiodestructive T-cell lymphoma [3].

Here we present a severe and devastating case with a very rare variant of LyP type E, which is, to our knowledge, the first case successfully treated with IFN alfa-2a.

## 2. Case Report

**Figure 1:**
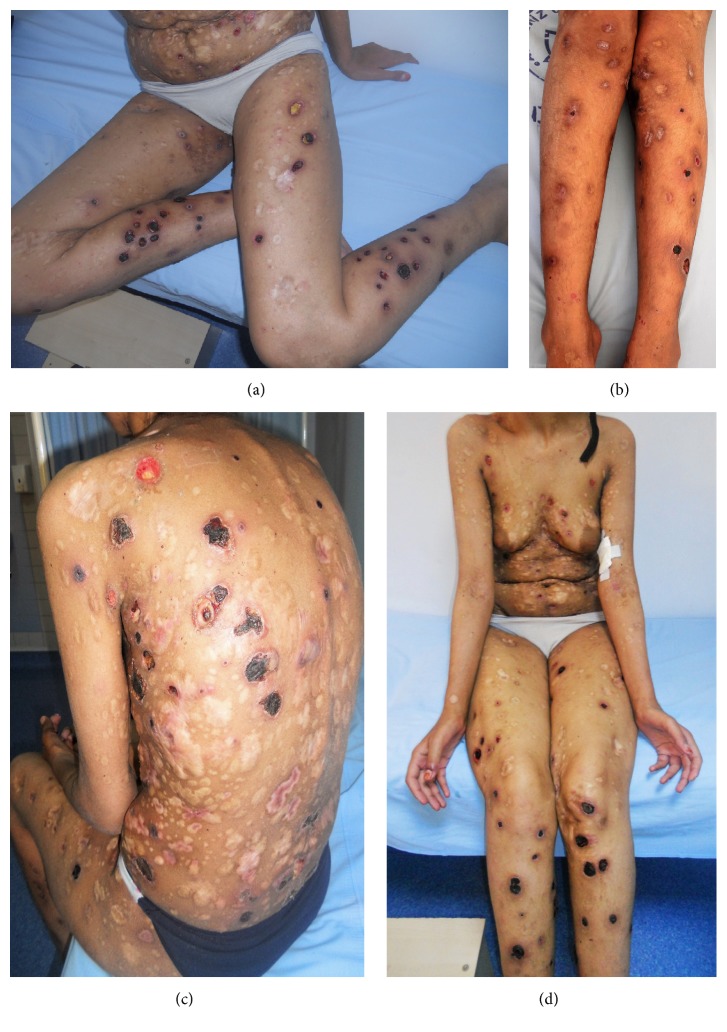
(a, b, c, d) Erythematous papules, plaques, hemorrhagic ulcerations, and atrophic scars in different sizes scattered all over the body.

**Figure 2:**
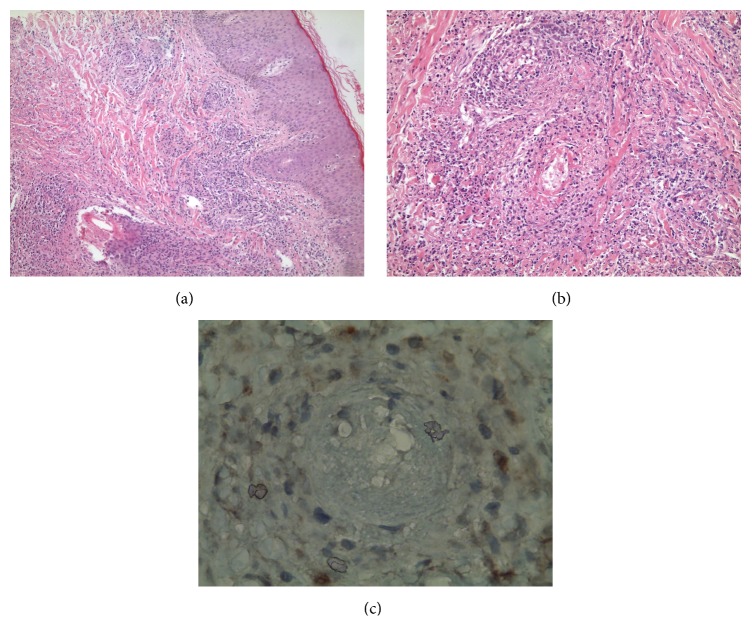
(a, b) Regular acanthotic epidermis, a dense dermal infiltrate of pleomorphic atypical lymphoid cells, and destruction of the blood vessels' walls by abnormal lymphocytes. (c) The cells were strongly positive for CD30.

A 18-year-old female presented to the outpatient clinic of dermatology with a 15-year history of waxing and waning course of erythematous papules, plaques, hemorrhagic ulcerations, and atrophic scars (Figures 1(a), 1(b), 1(c), and 1(d)). The lesions mostly started as painful erythematous papules and nodules on any site of the body following some constitutional symptoms such as fever and weakness. These lesions then quickly progressed to hemorrhagic deep ulcers resolving with depressed scar tissue either spontaneously or with nonspecific antibiotic therapies between 3 to 4 weeks. For the last 6-month duration, lesions appeared more frequently. She had no family history of similar lesions or other systemic diseases. On physical examination, there were multiple painful ulcers with necrotic base in different sizes scattered all over the body and numerous round atrophic scars (more than one hundred). She has no constitutional symptoms and palpable lymphadenopathy. The skin biopsy revealed regular acanthotic epidermis, a dense dermal infiltrate of pleomorphic atypical lymphoid cells, and destruction of the blood vessels' walls by abnormal lymphocytes (Figures 2(a) and 2(b)). Biopsy revealed abnormal lymphocytes which were predominantly positive for CD30 (Figure 2(c)) and mostly CD8+ lymphoid cells, especially angiocentric infiltrates. When we closely look at the entire infiltrate, which is both angiocentric and interstitial, an overall predominance of CD8+ cells over CD4+ cells was seen. CD20, CD56, and CD21 were negative. Perforin was negative and granzyme was focal positive. In situ hybridization for Epstein-Barr virus encoded RNA and Latent Membrane Protein 1 (LMP1) was negative. T-cell receptor (TCR) gene rearrangement could not be performed. Based on the clinical history, physical examination, and histological findings, diagnosis of LyP type E was established.

Hematologic laboratory and routine biochemistry workup showed no sign for systemic malignancy nor other systemic diseases. Chest/abdomen/pelvis computed tomography revealed irregular hyperdense areas on skin and subcutaneous tissue and multiple small (<1 cm) lymphadenopathies but neither hepatosplenomegaly nor other abnormalities were seen. On PET examination abdominal, axillary, inguinal, and cervical small hypermetabolic lymph nodes have been detected. Histopathological examination of a posterior cervical lymph node and bone marrow biopsy yielded unremarkable findings.

**Figure 3:**
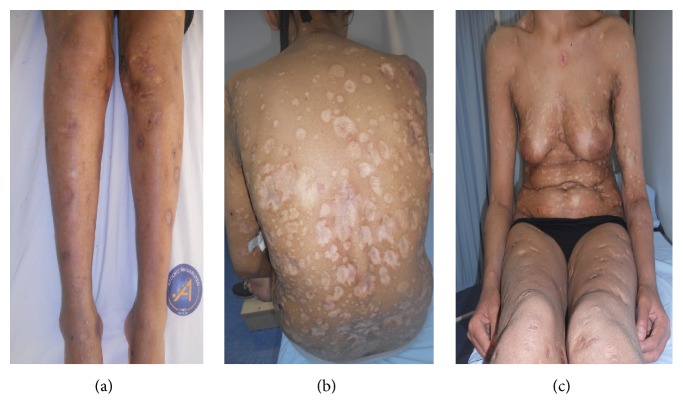
(a, b, c) All previous persistent lesions were healed completely and no new lesions were observed after two-month treatment with IFN alfa-2a.

As the patient has severe course with multiple devastating ulcerative lesions and frequent recurrences, we administered methotrexate with a dose of 15 mg per week. During 3-month follow-up, we observed a relative decrease in the appearance of new lesions. The dose of methotrexate was increased to 20 mg per week. After one month she had a severe recurrence of the disease while on treatment, and the dose was increased to 35 mg per week. However, the liver enzymes were elevated with this approach. The dose of methotrexate had to be discontinued. She was given IFN alfa-2a with a dose of 6 mU three times per week. After two-month treatment with IFN alfa-2a, she was finally feeling well and there were no new lesions and all previous persistent lesions were healed completely (Figures 3(a), 3(b), and 3(c)). A slight leucopenia was observed. Since clinical remission was achieved, the treatment schedule was switched to 6 mU IFN alfa-2a 2 times per week. For the time being the disease has been maintaining its remission status for six months.

## 3. Discussion

Clinical presentation of LyP is characterized by chronic recurrent, self-healing papulonodules which regress with scar formation. The duration of LyP is variable. It affects especially adults and usually persists with a range from several weeks to years with an excellent prognosis [4]. However, approximately 10% to 20% of patients with LyP may present with a lymphoproliferative malignancy such as CD30 anaplastic large cell lymphoma (ALCL), mycosis fungoides (MF), or Hodgkin disease [5, 6]. Thus, careful lifelong monitoring is required which allows early detection and treatment of potentially fatal lymphomas especially in children with LyP [7].

LyP type E is characterized with recurrent papulonodular lesions which rapidly turn into hemorrhagic necrotic ulcers and spontaneous regression by leaving a scar [2]. Up to date 18 cases were reported in the literature [3]. All of them had severe clinical features, with a typical progressive course. During follow-up, they all experienced several relapses like current patient [3].

None of the available treatment modalities affects the natural course of LyP. In most cases aggressive treatment is not required because of its favorable prognosis. Treatment can be considered in severe forms of LyP in which the size and number of lesions are extensive or when ulceration, scarring, and pruritus are prominent [4, 8]. For therapy various modalities have been used such as topical and systemic steroids, PUVA, methotrexate, bexarotene, and IFN alfa-2b. Low-dose methotrexate (5 to 25 mg weekly) is the most commonly reported single agent chemotherapy used to treat LyP. According to the results of retrospective analyses, it effectively suppresses the development of new lesions. However, the rapid relapse rate of 63% following methotrexate cessation often necessitates long-term maintenance therapy [4, 9]. Methotrexate was the first-line therapy in our case. However, although it initially suppressed the disease and provided a partial improvement in the clinical picture, it had to be discontinued because of the adverse effects to the liver.

LyP has been observed to progress into anaplastic large cell (CD30+) lymphoma, mycosis fungoides, and Hodgkin or non-Hodgkin lymphoma [8]. IFN alfa has been used successfully in these disorders which is thought to be due to effects on malignant clones [10]. In addition, Yagi et al. have examined the therapeutic efficacy of recombinant IFN-y in two patients with LyP [11]. They suggested that despite being speculative the mechanism of inhibition of CD30+ cell proliferation by IFN-y might involve two actions. First, IFN-y directly downregulates the transcription of cytokine mRNA by CD30+ cells which leads to inhibition of proliferation of these cells. In indirect action, IFN-y enhances mRNA transcription in inflammatory Thl cells, which might exert antitumor cell activity after activation [11]. In an open trial, researchers compared the clinical, histologic, and immunohistochemically features from a group of five patients receiving IFN subcutaneously three times per week with the same features from a group of six patients receiving conventional therapy, including photochemotherapy, antibiotics, topical corticosteroids, or surgery [12]. In the IFN group, four patients showed a complete remission, whereas one patient showed a partial remission within 6 weeks. These results indicate that the treatment with IFN of patients with LyP alters the clinical course of the disease with fewer side effects than previous regimens; however, short-term treatment does not induce sustainable remission. Therefore, prolonged treatment appears to be warranted for the long-term remissions in these patients [12]. Because of these reports about the value of interferon in the treatment of cutaneous lymphomas, and in some LyP cases, we decided to use IFN alfa-2a with a dose of 6 mU three times per week subcutaneously [8, 11–13].

In conclusion, we report a very severe case with recently described and rare type E variant of LyP. To our knowledge, this is first case of LyP type E successfully treated with IFN alfa-2a. Even though we think current case may provide some contribution, further knowledge and experiences are needed for morphological features and treatment of this new variant of LyP.
